# Serum Persistent Organic Pollutants and Duration of Lactation among Mexican-American Women

**DOI:** 10.1155/2010/861757

**Published:** 2010-06-30

**Authors:** Rosana H. Weldon, Monique Webster, Kim G. Harley, Asa Bradman, Laura Fenster, Mark D. Davis, Alan Hubbard, Dana B. Barr, Nina Holland, Brenda Eskenazi

**Affiliations:** ^1^Center for Children's Environmental Health Research, School of Public Health, University of California, Berkeley, CA 94704, USA; ^2^Division of Environmental and Occupational Disease Control, California Department of Public Health, Richmond, CA 94804-6403, USA; ^3^National Center for Environmental Health, Centers for Disease Control and Prevention, Atlanta, GA 30341-3717, USA; ^4^School of Public Health, Division of Biostatistics, University of California, Berkeley, CA 94720-7358, USA

## Abstract

*Background*. Research suggests that estrogenic endocrine-disrupting chemicals interfere with lactation. *Objectives*. (1) to determine if estrogenic persistent organic pollutants (POPs) are associated with shortened lactation duration; (2) to determine whether previous breastfeeding history biases associations. *Methods and Results*. We measured selected organochlorines and polychlorinated biphenyls (*p*, *p*′-DDE, *p*, *p*′-DDT, *o*, *p*′-DDT, *β*-hexachlorocyclohexane, hexachlorobenzene, and PCBs 44, 49, 52, 118, 138, 153, and 180) in serum from 366 low-income, Mexican-American pregnant women living in an agricultural region of California and assessed breastfeeding duration by questionnaires. We found no association between DDE, DDT, or estrogenic POPs with shortened lactation duration, but rather associations for two potentially estrogenic POPs with lengthened lactation duration arose (HR [95% CI]: 0.6 [0.4, 0.8] for *p*, *p*′-DDE & 0.8 [0.6, 1.0] for PCB 52). Associations between antiestrogenic POPs (PCBs 138 and 180) and shortened lactation duration were attributed to a lactation history bias. *Conclusion*. Estrogenic POPs were not associated with shortened lactation duration, but may be associated with longer lactation duration.

## 1. Introduction

Persistent organic pollutants (POPs) “persist in the environment, bioaccumulate, and pose a risk of causing adverse effects to human health and the environment” [1]. Pesticides such as dichlorodiphenyl trichloroethane (DDT), hexachlorocyclohexane (HCCH), and hexachlorobenzene (HCB) and industrial chemicals such as polychlorinated biphenyls (PCB) are POPs. Concerns regarding the effects of these chemicals on endocrine function in humans and wildlife led to the Stockholm Convention, a global treaty in which over 160 governmental parties agree to voluntarily eliminate or reduce the use of POPs. However, this agreement exempted uses of DDT for malaria control. Although chemical-free efforts are being endorsed, indoor spraying of DDT remains a primary means of controlling malaria-transmitting mosquitoes in developing countries [2].

For all infants, but particularly for those from impoverished regions (where use of DDT is more common), breastfeeding is the optimal source of nutrition because it helps fight infections, including malaria, reduces dehydration, and is less expensive than formula feeding [3]. Breastfeeding has additional benefits for the mother and child, including postpartum uterine contractions, lactational amenorrhea, and increased bonding [4]. WHO recommends exclusive breastfeeding for infants during the first six months of life and continued breastfeeding with complementary foods up to two years and beyond [5]. Exclusive breastfeeding rates around the world are generally low with fewer than 35% of children receiving only breast milk for the first four months of life [5].

In the United States (U.S.), the most commonly reported barriers to breastfeeding initiation and duration among low-income mothers include concerns over whether the baby is getting enough to eat, concerns about breastfeeding in public, and fear of difficulty or pain during breastfeeding [6, 7]. One study reported that Hispanic U.S. mothers were more likely to report milk insufficiency and infant breast refusal as the primary reason for cessation of breastfeeding compared to African-American or white mothers [7]. 

Synthetic estrogens such as hormonal contraceptives, smoking and *p*,*p*′-dichlorodiphenyl dichloroethylene (*p*,*p*′-DDE), an organochlorine (OC) metabolite of DDT, have been associated with shortened lactation duration, presumably due to effects on milk supply [8–12]. However, the association between *p*,*p*′-DDE and duration of lactation has been questioned by investigators who could only reproduce the finding among women who had previously lactated [10, 12].

Breastfeeding is a major excretory route for POPs and the longer a woman breastfeeds the more she reduces her body burden of POPs [13]. Women tend to breastfeed subsequent children for approximately the same time as previous children [14, 15]; therefore, women who breastfed previous children for a short time may have higher levels of POPs, but will breastfeed subsequent children for a short time, possibly biasing studies examining the effects of POPs on lactation duration [9, 10, 12]. Thus, true relationships between POPs and length of lactation, should be found in women who had not lactated previously.

In addition, women are likely exposed to a mixture of several endocrine-disrupting POPs that may also affect breastfeeding duration. DDT and DDE, and other chemicals including *β*-HCCH, HCB, and individual congeners of PCBs, have been evaluated by animal or *in vitro* studies for their ability to bind to estrogen or androgen receptors and their ability to elicit or inhibit an endocrine response [16–20].

In this study, we examine the relationships between concentrations of persistent organic pollutants measured in the serum of Mexican-American women living in an agricultural region and duration of lactation using data collected from the Center for the Health Assessment of Mothers and Children of Salinas (CHAMACOS)—a longitudinal birth cohort that began in 1998. We first summarize the available literature on the endocrine-disrupting activity of commonly-detected POPs and categorized them by activity. We then determine the associations between POPs and duration of lactation individually and by these categories. Based on Rogan and Gladen's [9] hypothesis that the estrogenic activity of *o*,*p*′*-*DDT, *p*,*p*′-DDT and *p*,*p*′*-*DDE may shorten lactation duration, we expected POPs with estrogenic activity to be associated with shortened lactation duration while POPs with antiestrogenic endocrine-disrupting activity to be associated with lengthened lactation duration. Since the CHAMACOS dataset contains information on duration of breastfeeding for all previously breastfed children, we also investigated the potential bias introduced by breastfeeding history.

## 2. Materials and Methods

### 2.1. Study Population

The study population was drawn from CHAMACOS, a longitudinal birth cohort comprised of 601 pregnant women [21]. The purpose of this cohort is to study the effects of pesticides and other environmental exposures in pregnant women and their children who were born in the Salinas Valley of California, an agricultural community. Women were eligible to participate if they were over 18 years of age, English- or Spanish-speaking, less than 20 weeks gestation at enrollment, Medi-Cal eligible, and planning to deliver at Natividad Medical Center. Of the 601 women who were enrolled in this study, 526 delivered liveborn singletons. We restricted the analyses to those who initiated breastfeeding (*n* = 498) and who provided some information on the duration of lactation (*n* = 487). Of these, 366 women had adequate volume of serum drawn near 26 weeks of gestation for measurement of concentrations of persistent organic pollutants (POPs). Demographic characteristics in this subset of women did not differ from the larger cohort.

### 2.2. Procedure

CHAMACOS mothers were interviewed by bilingual, bicultural staff about their demographic characteristics and pregnancy and breastfeeding histories. Information was collected on maternal age, parity, body mass index, maternal education and work status, years residing in the United States, marital status and family income. Women were also asked how long they had breastfed all previous children. At delivery, and at 6-, 12-, 24- and 42-months postpartum, women were asked if they were currently breastfeeding their child, and if not, the child's age in months or weeks when they completely stopped breastfeeding. Information on prenatal care and delivery was abstracted from medical records by a nurse.

Women provided written consent and all study protocols were approved by the Committee for the Protection of Human Subjects at the University of California, Berkeley with collaboration from the Institutional Review Board at the Centers for Disease Control and Prevention (CDC).

### 2.3. Sample Collection and Laboratory Analyses

Serum samples were collected and processed near the end of the second trimester of pregnancy (27.3 ± 3.1 weeks gestation), as previously described in [22]. The OC pesticides and PCBs (*p*,*p*′-DDT, *o*,*p*′-DDT, *p*,*p*′-DDE, HCB, *β*-HCCH, and PCBs 44, 49, 52, 118, 138, 153, and 180) were chosen for data analysis because they were thought to affect the estrogenic or androgenic hormone systems, have been examined in previous literature on duration of lactation, or are detected most frequently in human populations. OC pesticides or degradates and PCBs were analyzed by the CDC in Atlanta, GA using a validated method [22, 23]. Serum samples (1 g) were fortified with isotopically labeled standards of each analyte and dispersed with Hydromatrix in pressurized fluid cells to facilitate extraction. Samples were lyophilized and Florisil was added to the bottom of the cells. Pressurized fluid extraction was performed using 20% dichloromethane in hexane at 100°C and 1500 psi. Gel permeation chromatography was used to further purify the extracts prior to analysis by gas chromatography high-resolution mass spectrometry. Each run of approximately 20 unknown samples contained additional quality control and blank samples. Isotopic dilution calculations were used to quantify concentrations of OC pesticides and PCBs in all samples. The mean (SD) limits of detection (LODs) in pg g^−1^ serum were as follows: 3.0 (2.1) for *p*,*p*′-DDE; 1.6 (1.8) for *p*,*p*′-DDT; 1.3 (2.2) for *o*,*p*′-DDT; 0.8 (1.0) for HCB; 1.6 (0.7) for *β*-HCCH; 2.9 (1.3) for PCB 44; 2.3 (1.1) for PCB 49; 2.6 (1.2) for PCB 52; 1.4 (0.6) for PCB 118; 1.2 (0.4) for PCB 138; for 1.2 (0.5) PCB 153; and 1.2 (0.5) for PCB 180. Levels below the LOD were assigned the value of LOD/2 [24, 25]. Detection frequencies ranged from 96%–100% and have been detailed elsewhere [22, 26]. Sample sizes among POPs varied because some measures failed to meet quality control criteria. 

Enzymatic lipid analysis (Roche Chemicals, Indianapolis, IN, U.S. A.) was performed for each sample to measure total cholesterol and triglycerides from which total lipids were calculated using methods reported by Phillips et al. [27]. Lipid-adjusted concentrations in nanograms per gram serum lipid (ng g^−1^ lipid) were used for all statistical analyses.

### 2.4. Endocrine Disruption Categories

OC pesticides and PCBs were categorized as potentially estrogenic, antiestrogenic, androgenic and/or antiandrogenic (Table 1) based on *in vitro *and animal studies published and identified using PubMed and Google Scholar [28, 29]. Search terms consisted of the combinations of the chemical names with estrogen, androgen, endocrine, or enzyme. Chemicals with conflicting data were placed in multiple categories.

### 2.5. Statistical Methods

All data analyses were performed using Stata 10 for Windows [30]. Independent variables were lipid-adjusted maternal serum concentrations of POPs. Since exposures to these persistent chemicals may arise by common pathways, a correlation matrix of POPs was constructed. The POP concentrations were treated two ways: as categorical variables (in quartiles) and continuously (log _10_ -transformed). Kaplan-Meier plots were used to estimate median durations of lactation for each quartile of exposure. Both crude and adjusted Cox proportional hazards models were used to estimate the instantaneous probability of weaning given that weaning had not yet occurred. Some women were lost to follow-up prior to weaning or were still breastfeeding at the time of the 42-months postpartum assessment (14%) and were right censored at the time of the last recorded information (38 were between the hospital delivery visit and six months, 8 were lost from six up to 12 months, and 4 were censored at 12 months and beyond). Fractional polynomial regression was used to assess whether linear models using continuous concentrations fit the data as well or better than models with one or two additional terms (knots) that allow for a curved relationship; for each POP, the linear model was satisfactory [31]. Only continuous models are reported since results using quartiles produced similar results.

Demographic and maternal characteristics that were associated with the exposures [22] or with duration of lactation [9–11, 32] in previous literature were considered potential confounders. These variables were categorized as indicated in Table 2 and included mother's age at delivery, previous lactation history, maternal education, maternal work status during pregnancy, mode of delivery, marital status, and maternal body mass index [33]. A measure of social class was derived from the year 2000 federal poverty guidelines [34]. Models included and excluded years residing in the United States since this variable may be a potential confounder [22, 32] but it may also be a proxy for exposure for some chemicals. In addition, we considered this variable as an effect modifier, particularly for the DDT/DDE isomers since women with high DDT/DDE concentrations may have moved to the US more recently from Mexico and may also breastfeed longer due to cultural influence.

Covariates were chosen for adjusted models using a manual backward elimination strategy and were retained if they were associated with the outcome and the exposures using Wald tests for continuous or dichotomous covariates and F-tests for categorical covariates. Since the use of lipid-adjusted POP concentrations may bias linear associations with epidemiological outcomes [35], we reanalyzed all models using nonlipid-adjusted POP concentrations, while including lipid concentrations as a covariate. Coefficients and the resulting inference were nearly identical using both approaches; thus, we have only presented data using lipid-adjusted POP concentrations. Similarly, imputing values for concentrations that are <LOD may also bias results [36]; therefore, we reanalyzed all models using (i) a value of 0.001 ng/g lipid for all observations that were <LOD and (ii) the LOD itself for all observations that were <LOD. Both strategies resulted in nearly identical coefficients, hazard ratios, and inference to models using the LOD/2 imputation strategy; thus, models using the original LOD/2-imputed values are presented.

Previous researchers have suggested that associations between POPs and lactation duration may be biased by previous lactation [9–12]. For example, if a woman's serum concentration of *p*,*p*′-DDE declines over the course of lactation and this woman chooses to breastfeed a previous child for a short time, her serum concentration of *p*,*p*′-DDE may be higher than a woman who breastfed for a long time. Additionally, since women tend to breastfeed subsequent children for a similar duration [14, 15], an association between higher *p*,*p*′-DDE concentration and shortened lactation duration may arise in women who previously breastfed for a short time. This bias is not present among women who never previously lactated. Thus, we investigated this bias by constructing adjusted Cox models including the cross-product of the variable for whether a woman previous lactated (Yes/No) and continuous log _10_ -transformed POP concentrations to assess interaction. Stratified results were derived from interaction models. An interaction term with a significance level of 0.15 was considered sufficiently statistically significant to pursue follow-up analyses. 

Within the group of women who previously breastfed, we also controlled for average length of previous lactation. We summed lactation duration for all previously born children (excluding the CHAMACOS child) and averaged the total duration by the number of previous children to summarize a mother's previous breastfeeding habits. We correlated the average previous lactation duration by the duration of breastfeeding for the CHAMACOS index child. We then performed Cox models among the women who breastfed previous children using continuous log_10_-transformed concentrations of the POPs while adjusting for average previous breastfeeding duration and other covariates. 

Lastly, we used principal components analysis to summarize all of the chemicals within an endocrine-disrupting activity category as identified in Table 1. Separate principal components analyses were performed for each of the four categories and the first principal component in each analysis was used as the summary variable of the entire endocrine-disrupting activity category. Chemicals which fell under multiple categories were included in each of their respective principal component analyses. Values that were missing for reasons other than below the detection limit (e.g., if the measured concentration did not meet quality control criteria) were imputed with the median value for the respective chemical in order to retain the sample size. The first principal component summary variable, which explained 36% of the total variance for chemicals in the estrogenic category, 66% for the antiestrogenic category, 60% for the androgenic category, and 40% for the antiandrogenic category, was then used in separate Cox models for each category including an interaction term (the cross-product of the summary variable and previous lactation); models were adjusted for maternal age at delivery, mother's years of residence in the United States and marital status. In addition, we used the concentrations imputed with the median value for missing observations to build a comprehensive model including all POPs as explanatory variables in the same model as well as maternal age as a covariate and stratified these models on previous breastfeeding.

## 3. Results

The demographic and pregnancy characteristics of our population are summarized in Table 2. In general, our population was comprised of nonsmoking women (99%) who were born in Mexico (90%), Spanish-speaking (90%), and living within 200% of the poverty level (99%). The majority of women had lived in the United States for five or fewer years (57%) and most women (62%) were overweight or obese prior to pregnancy. Additionally, over 40% of women worked in agriculture during pregnancy. Women in this study were young (mean 26 ± 5 years) with 78% aged 18–29 years. About 65% of women had a previous birth and 57% had previously breastfed. About 7% of the index children were born preterm (<37 weeks) and 3% were born of low birth weight (<2500 g). One-third of women breastfed the index child for <3 months, 39% for 3 to 11.9 months, and 28% for ≥12 months. Median duration of lactation was 6 months (Table 3). Median length of lactation among women with no history of breastfeeding was nearly half that of women who breastfed previous children, (4 versus 7 months, resp.). Among women who had previously breastfed, average duration of lactation in the past was moderately correlated with duration of lactation for the CHAMACOS child (*ρ* = 0.46, *P* < .0001).

Median serum levels in ng g^−1^ lipid were 1064 for *p*,*p*′-DDE, 12.7 for *p*,*p*′-DDT, 1.3 for *o*,*p*′-DDT, 39.3 for *β*-HCCH, 63.7 for HCB, 2.8 for PCB 44, 1.9 for PCB 49 3.7 for PCB 52, 3.5 for PCB 118, 2.4 for PCB 138, 5.2 for PCB 153, and 1.4 for PCB 180. Maternal serum concentrations of DDT and DDE isomers were correlated with each other (*ρ* *0* *.*8-0.9, *P* < .0005). Chemicals with primarily antiestrogenic activity, HCB, and PCBs 118, 138, 153, and 180, were also correlated with each other (*ρ* 0.3–1.0, *P* < .0005). As expected, since they are less persistent, PCBs 44, 49, and 52 were not correlated with PCBs 138, 153, and 180 (*ρ* < 0.05, *P* > .4 for all correlations), but were highly correlated among each other (*ρ* ~ 1.0, *P* < .0005). *p*,*p*′-DDE was correlated with PCB 180 (*ρ* 0.2, *P* = .002). Median concentrations of POPs (with the exception of PCB 52) were lower in women who had previously breastfed than women who had not (data not shown).

Table 3 shows the median duration of lactation for each quartile of exposure to each POP derived from the unadjusted Kaplan-Meier plots. Contrary to previous literature, median duration of lactation was longer among women in the highest quartile compared to the lowest for *p*,*p*′-DDE, *p*,*p*′-DDT, *o*,*p*′-DDT and for most of the potentially estrogenic chemicals. However, median duration of lactation was lower in the highest quartile than in the lowest for some of the potentially antiestrogenic chemicals (HCB, and PCBs 138, 153 and 180, but not PCB 118). Among women who had not previously breastfed, for all POPs except PCB 118 and 180, those in the highest quartile of exposure breastfed longer than those in the lowest quartile. 

Adjusted Cox models supported the findings of the unadjusted Kaplan-Meier medians and showed that most of the potentially estrogenic POPs (*p*,*p*′-DDE, *p*,*p*′-DDT, *o*,*p*′-DDT, PCB 49 and PCB52) were associated, albeit nonsignificantly, with decreased hazard of weaning (i.e., longer duration of lactation), while antiestrogenic POPs (HCB, PCB 138, PCB 153 and PCB 180) were borderline or significantly associated with an increased hazard of weaning (i.e., shortened duration of lactation) (Table 4). However, these results differed by previous history of lactation. We hypothesized that estrogenic chemicals such as *p*,*p*′-DDE would cause shortened lactation duration in women who had not breastfed prior children if the relationship were driven solely by chemical exposures rather than previous lactation history. However, we found increasing concentrations of *p*,*p*′-DDE were associated with a decreased hazard of weaning (i.e., breastfed longer) (HR [95% CI] = 0.6 [0.4, 0.8]) among women who did not previously breastfeed, while no statistically significant association was observed among women who previously breastfed (HR [95% CI] = 1.1 [0.8, 1.4]; *P*_interaction_ = .01). When we adjusted for average duration of lactation for previously born children among women who had breastfed, other potentially estrogenic POPs, PCBs 44, 49, and 52, also became significantly or borderline protective against weaning (i.e., associated with longer lactation) (HR [95% CI] = 0.7 [0.4, 1.0], 0.5 [0.3, 0.8], and 0.7 [0.4, 1.0], resp.).

Conversely, we considered that higher concentrations of antiestrogenic chemicals might result in longer lactation duration among women who did not previously breastfeed. However, we found that increasing concentrations of both PCBs 138 and 180 were not associated with lactation duration among women who did not breastfeed previously, but among women who breastfed previous children, increasing concentrations of both congeners were associated with an increased hazard of weaning (i.e., breastfed shorter) (HR [95% CI] = 2.2 [1.2, 4.0] and 2.1 [1.2, 3.9], resp.;*P*_interaction_ = .11). After adjusting for average breastfeeding duration among women who breastfed previous children, PCB 180 (HR [95% CI] = 2.0 [1.1, 3.8]), but not 138 (HR [95% CI] = 1.7 [0.9, 3.2]), remained associated with an increased hazard of weaning.

We considered the relation of DDT/E and length of lactation stratified by years in the US. Overall, results were similar across stratum of duration of residence (≤1, 2–5, 6–10, ≥11 years) in the US. However, we observed that among women who previously lactated, but not among those who did not, there was a significant decreased hazard of weaning (i.e., longer lactation) associated with DDT and DDE serum levels among the women who had been in the US the shortest and the longest, but not for the two middle groups (data not shown).

When we used summary variables generated by principal components analysis as our main independent variables (Table 5), we found that the antiestrogenic group of chemicals was associated with shortened lactation duration, but only among women who breastfed previous children (HR [95% CI] = 1.1 [1.0, 1.3]). Variables that summarized estrogenic, androgenic, and antiandrogenic chemicals were not associated with lactation duration. Our comprehensive models including all POPs and maternal age as a covariate showed that only PCB 52 was associated with longer lactation duration (HR [95% CI] = 0.86 [0.77, 0.98]). Upon stratifying by previous breastfeeding, we found that this association was only among women who never previously breastfed (HR [95% CI] = 0.76 [0.61, 0.96]).

## 4. Discussion

We examined whether individual endocrine-disrupting OC pesticides and PCB congeners were associated with duration of lactation in Mexican-American women residing in an agricultural area. Contrary to previous reports, we found that increasing *p*,*p*′-DDE maternal serum concentrations were associated with *increased* duration of lactation among women who had not previously breastfed; we found no association in women who had breastfed previous children. In addition, we found increasing concentrations of potentially antiestrogenic POPs were associated with reduced duration of lactation, but only among women who had breastfed previous children. The concentrations of OCs in this population are higher than the US population of women of child-bearing age for *p*,*p*′-DDE, *p*,*p*′-DDT, HCB and *β*-HCCH (US Medians = 174, 8, 40, and 5 ng g^−1^ lipid (LOD imputed), resp.), but lower for PCBs (US Medians = 4, 4.6, 14, 20, and 10 ng g^−1^ lipid (LOD imputed) for PCBs 52, 118, 138, 153, and 180, resp.) [22, 55, 56].

All of the associations between POPs and shortened lactation duration in our study were only found among women who breastfed previous children. These associations can be attributed to lactation history bias because (1) previous breastfeeding duration (averaged per child) was correlated with duration of breastfeeding the index child in our dataset; (2) with the exception of *o*,*p*′-DDT, all POPs examined in this study were negatively associated with months of previous lactation, although only PCBs 138, 153, and 180 were statistically significant. A true association between an endocrine-disrupting chemical and shortened lactation duration should be observed within the group of mothers who had not previously lactated. 

Our results are not consistent with the four previous studies of DDE. Our sample size of ~366 women is somewhat larger than the Durango [10] and Michigan [11] studies (*N* = 229 and 310, resp.), but smaller than the Chiapas [12] and North Carolina [9] studies (*N* = 750 and 858, resp.). Assuming that lipid-adjusted milk concentrations are equal to 1.74 times lipid-adjusted serum concentrations [57], our median *p*,*p*′*-*DDE concentration of 1060 ng g^−1^ lipid is similar to the two US populations (~1000 ng g^−1^ lipid in Michigan & 1400 ng g^−1^ lipid in North Carolina), but lower than the two Mexican populations (2700 ng g^−1^ lipid in Chiapas and 3400 ng g^−1^ lipid in Durango). Of these four previous studies, Rogan et al. and Karmaus et al., with concentrations similar to those in the present study, found shortened lactation duration with increasing concentrations of DDE among women who did not breastfeed previously [9, 11]. In contrast, we found that increasing concentrations of DDE were associated with longer duration of lactation among women who did not breastfeed previously.

Our study population is comprised of a migrant Mexican-American population who has spent varying amounts of time in the US. Residual confounding by acculturation (despite controlling for years of residence in the US) could explain the disparity between our results and those of previous studies, in that fewer years of residence in the US is associated with higher DDT/E concentrations (due to Mexico's relatively recent use of DDT compared to the US), but longer duration of lactation (due to cultural influence). However, we observed a similar relationship of longer duration of lactation associated with DDT and DDE serum concentrations even within the group that had been in the US ≤1 year. Although we did not adjust for multiple hypothesis testing, the *P*-value for the association between *p*,*p*′-DDE and lengthened lactation duration among women who never previously lactated was .002. This *P*-value is lower than a Bonferroni significance level adjusted by 12 chemicals (*P*_Bonferroni_ = .004), which suggests that this association may not be a chance finding. However, there may be other confounding factors that we could not control for due to lack of information including spouse, family and friends' attitudes and support of breastfeeding, or other social factors that may influence breastfeeding duration. 

Our findings are also not consistent with previous studies which found no association between PCBs and lactation duration regardless of lactation history [9, 11]. We report associations between PCB 138 and PCB 180 with shortened lactation duration, but confined to women who had prior breastfeeding history. Thus, we attributed these findings to the bias introduced by previous breastfeeding. In an attempt to control this bias among women who previously breastfed, we added a covariate for average duration of lactation of previous children to our models and found that PCB 180 remained associated with shortened lactation duration and that PCBs 49 and 52 became significantly associated with longer breastfeeding duration. PCB 52 was also associated with longer lactation duration among women who had never previously breastfed in our comprehensive model including all POPs, thus, adding to the evidence that PCB 52, an estrogenic chemical, may be associated with longer lactation duration. If the associations for PCBs 49 and 180 had been observed both among women who had never previously lactated and women who previously lactated (controlling for previous lactation duration), we would be more inclined to conclude that there may be an association between PCB 180 (an antiestrogenic chemical) and shortened lactation duration as well as an association between PCB 49 (an estrogenic chemicals) with longer lactation duration. However, given the inconsistencies of these results in those with and without a history of lactation, relationships for PCBs 49 and 180 may be spurious.

Previous studies examining relationships between POPs and duration of lactation have reported only on associations with *p*,*p*′-DDE or total PCB concentrations [9–12]. No previous studies have examined effects of several POPs or determined whether chemicals with similar endocrine-disrupting activity affect duration of lactation similarly. In this study, we attempted to classify analytes based on toxicologic mechanism. We hypothesized that if chemicals with similar toxicologic profiles affected lactation in the same manner, this would strengthen the biological plausibility of the results. This approach of categorizing chemicals based on biological mechanism has been previously applied in studies of cancer [58] and thyroid function [26]. However, there are limited data from *in vitro* and animal studies that allow for the categorization of the endocrine-disrupting potential of specific chemicals, the results of these studies are often conflicting [59, 60], and some chemicals have multiple mechanisms. Using principal components models we could not account for the relative potency of a chemical or whether chemicals act synergistically or antagonistically, but our findings largely supported the results generated from separate models for each chemical. Only the antiestrogenic category was associated with shortened lactation duration, but this finding was also attributable to the lactation history bias.

Our study has some limitations. Although previous studies have postulated that breastfeeding initiation may be more sensitive to effects of endocrine disruptors [12], we could not examine this association because the majority of women in the CHAMACOS cohort (97%) initiated breastfeeding. However, among 11 women who did not initiate breastfeeding and who had blood POP measurements, the median concentration of *p*,*p*′-DDE was 880 ng g^−1^ lipid. This value fell into the second quartile of exposure and suggests that in our population, initiation may not be affected by *p*,*p*′-DDE exposure. In addition, there may be biases introduced by self-reported data. In our study, women were asked to report the age of their infant (in months or weeks) at the time that they completely stopped breastfeeding. Such self-reported data can lead to misclassification errors. However, since we asked the mother for this information at 6-, 12-, 24- and 42-months postpartum (within 6–18 months of her weaning), our data are likely accurate. Women did not know their exposure status at the time that they were interviewed, which would minimize recall bias. Although we have no evidence of reporting bias, it is possible that women may have reported longer lactation duration than actually occurred because they perceived breastfeeding as more beneficial to their children. Lastly, although POP concentrations measured at approximately the 26th week of gestation are well correlated with a subset of concentrations measured near delivery (*n* = 20, *ρ* > 0.7 for most chemicals), we do not know the critical exposure window for duration of lactation and cannot be certain that concentrations measured at approximately the 26th week of gestation represent this critical exposure window [61]. Thus, results may be different if all concentrations were measured closer to delivery or during the postpartum period.

## 5. Conclusions

Our findings do not support previous associations between *p*,*p*′-DDE and shortened lactation duration nor do we find any associations between shortened lactation and any estrogenic POP. Instead, we found associations for two potentially estrogenic POPS, *p*,*p*′-DDE and PCB 52 and lengthened lactation duration in women who had not lactated previously. The associations for *p*,*p*′-DDE and lengthened lactation duration were observed even among those who had resided in the US for one or fewer years. We also found shortened lactation duration with antiestrogenic POPs among women who breastfed previously born children, but these associations may be spurious since they were not seen among women who did not breastfeed previous children.

## Figures and Tables

**Table 1:** Endocrine disruption categories based on published literature.

Estrogenic	Antiestrogenic	Androgenic	Antiandrogenic
*p*,*p*′-DDE [17]	HCB [37–40]	HCB [18]	*p*,*p*′-DDE [17, 41]
*p*,*p*′-DDT [17, 42]	PCB 118 [43, 44]	PCB 118 [45]	*p*,*p*′-DDT [17]
*o*,*p*′-DDT [17, 42, 46, 47]	PCB 138 [43, 44, 48, 49]		*o*,*p*′-DDT [17]
*β*-HCCH [16, 17, 46, 50]	PCB 153 [43, 48, 49, 51]		HCB [18]
PCB 44 [19, 44]	PCB 180 [43, 48, 49]		PCB 118 [45]
PCB 49 [19, 44]			PCB 138 [49]
PCB 52 [44, 48, 52]			
PCB 153 [53, 54]			

Abbreviations: dichlorodiphenyldichloroethylene (DDE), dichlorodiphenyltrichloroethane (DDT), hexachlorocyclohexane (HCCH), hexachlorobenzene (HCB), polychlorinated biphenyl (PCB).

**Table 2:** Characteristics of CHAMACOS participants (*N* = 366), Salinas Valley, California, 1999-2000.

	*n*	(%)
Lactation Duration (months)		
<3	120	(32.8)
3–12	144	(39.3)
12+	102	(27.9)
Mother's Age at Delivery (years)		
18–24	162	(44.4)
25–29	124	(34.0)
30–34	49	(13.4)
35–45	30	(8.2)
Parity		
0	130	(35.5)
1	116	(31.7)
2+	120	(32.8)
Breastfed Previous Children		
No/Not applicable	155	(42.7)
Yes	208	(57.3)
Mother's Education		
6th grade or less	159	(43.4)
7–12th grade	136	(37.2)
High school graduate or more	71	(19.4)
Mother's work status		
Did not work	139	(38.4)
Some field work	108	(29.8)
Some agricultural work	42	(11.6)
Other work only	73	(20.2)
Body Mass Index (kg m^−2^)		
Underweight (<18.5)	3	(0.9)
Normal (18.5–25)	133	(37.7)
Overweight (25–30)	140	(39.7)
Obese (>30)	77	(21.8)
Years residing in United States		
≤1	102	(27.9)
2–5	108	(29.5)
6–10	81	(22.1)
11+	75	(20.5)
Marital Status		
Single	70	(19.1)
Married/Living as married	296	(80.9)
Cesarean Delivery		
No	277	(75.7)
Yes	89	(24.3)
Family Income		
At or below poverty level	216	(71.8)
Poverty-200%	83	(27.6)
Above 200% Poverty level	2	(0.7)

**Table 3:** Median duration of lactation from Kaplan-Meier plots by quartiles of persistent organic pollutant concentrations for all participants and stratified by breastfeeding history, Salinas, California, 1999-2000.

						Median duration of lactation (months)		
	*N*	Quartile	*n*	Exposure Range^a^ (ng g^−1^ lipid)	Exposure Median (ng g^−1^ lipid)	All Participants	Did not Breastfeed Previously	Breastfed Previously
Overall						6.0	4.0	7.0
*p*, *p*′-DDE^b,e^	366							
		1	92	48.8–568.9	406.8	5.0	2.0	7.0
								
		2	91	572.2–1059.9	808.8	6.0	3.0	7.5
		3	92	1067.6–2697.9	1531.5	6.0	6.0	5.0
		4	91	2801.6–159303.3	7022.2	8.0	5.0	10.0
*p*, *p*′-DDT^b,e^	366							
		1	92	1.6–7.0	4.7	5.0	3.0	5.5
		2	91	7.1–12.7	9.6	7.0	5.0	10.0
		3	92	12.8–35.5	19.0	6.0	3.0	6.5
		4	91	39.3–33174.0	201.8	7.0	5.0	8.0
*o*, *p*′-DDT^b,e^	364							
		1	91	0.1–0.7	0.5	5.0	2.0	7.0
		2	91	0.7–1.3	0.9	6.5	7.0	5.5
		3	91	1.3–3.1	1.8	7.0	4.5	9.0
		4	91	3.1–1878.1	8.1	6.0	4.0	7.0
*β*-HCCH^b^	364							
		1	91	0.1–19.2	7.9	6.5	3.5	9.5
		2	91	19.2–38.9	28.8	7.0	2.7	9.0
		3	91	39.6–77.1	50.4	5.0	3.0	7.0
		4	91	77.2–2491.6	119.5	6.0	6.0	6.0
HCB^b,c,d,e^	366							
		1	92	7.5–38.8	26.7	6.0	3.0	8.0
		2	91	39.7–63.7	50.8	7.0	2.3	11.0
		3	92	63.7–107.6	81.0	6.0	5.5	8.0
		4	91	109.5–710.1	161.1	4.5	3.5	5.0
PCB 44^b^	301							
		1	82	0.2–1.4	0.8	5.0	3.0	7.0
		2	81	1.4–2.8	2.0	7.0	5.0	12.0
		3	82	2.8–4.4	3.4	6.0	3.5	7.0
		4	81	4.4–11.4	5.7	6.0	4.0	8.0
PCB 49^b^	317							
		1	86	0.1–0.9	0.5	3.0	2.7	6.0
		2	86	0.9–1.9	1.4	7.5	4.0	11.0
		3	86	1.9–2.8	2.3	6.0	5.0	6.5
		4	85	2.9–7.9	3.8	7.0	4.0	9.0
PCB 52^b^	334							
		1	91	0.02–2.0	1.2	5.0	3.0	7.0
		2	90	2.0–3.7	2.8	5.5	5.0	6.0
		3	91	3.7–5.3	4.4	6.0	3.5	6.5
		4	90	5.4–12.4	7.2	7.0	5.5	7.0
PCB 118^c,d,e^	342							
		1	86	0.1–2.5	1.9	5.0	3.0	10.5
		2	85	2.5–3.5	3.0	7.0	3.5	7.5
		3	86	3.5–4.8	4.1	6.0	6.0	9.0
		4	85	4.8–25.1	6.5	5.0	3.0	6.0
PCB 138^c,e^	334							
		1	84	0.2–1.6	1.3	7.0	3.0	12.0
		2	83	1.6–2.4	1.9	5.0	3.0	8.0
		3	84	2.4–3.6	2.8	7.0	4.0	9.0
		4	83	3.6–30.9	5.2	5.0	5.5	4.5
PCB 153^b,c^	348							
		1	87	0.3–3.6	2.9	7.0	3.5	11.0
		2	87	3.6–5.2	4.3	6.0	5.0	8.0
		3	87	5.2–8.0	6.2	5.0	4.0	6.0
		4	87	8.0–95.7	11.6	6.0	5.5	6.0
PCB 180^c^	284							
		1	71	0.3–0.9	0.7	6.0	5.0	8.5
		2	71	0.9–1.4	1.1	7.0	3.0	9.0
		3	71	1.4–2.3	1.6	5.8	5.0	6.0
		4	71	2.3–30.0	3.3	4.5	4.5	4.5

^a^Maternal serum drawn at approximately 26 weeks gestation, values below the limit of detection (LOD) were assigned the value of LOD/2; ^b^Estrogenic category; ^c^Antiestrogenic category; ^d^Androgenic category; ^e^Antiandrogenic category.

Abbreviations: Persistent organic pollutant (POP), sample size (*N*), sample size of subset (*n*), dichlorodiphenyldichloroethylene (DDE), dichlorodiphenyltrichloroethane (DDT), hexachlorocyclohexane (HCCH), hexachlorobenzene (HCB), polychlorinated biphenyl (PCB).

**Table 4:** Relationships between log-transformed persistent organic pollutant (POP) concentrations in maternal blood drawn at 26 weeks gestation and hazard of weaning.

				Interaction of breastfeeding history and POPs		
		Unadjusted	Adjusted	Did not Breastfeed Previously	Breastfed Previously	
	*N*	HR (95% CI)	HR (95% CI)	HR (95% CI)	HR (95% CI)	*P*_interaction_^f^
*p*, *p*′-DDE^a^	366	0.9 (0.7, 1.1)	0.9 (0.7, 1.1)	0.6 (0.4, 0.8)**	1.1 (0.8,1.4)	.01*
*p*, *p*′-DDT^a^	366	0.9 (0.8, 1.0)	0.9 (0.8, 1.1)	0.8 (0.7, 1.1)	1.0 (0.8, 1.2)	.37
*o*, *p*′-DDT^a^	364	0.9 (0.7, 1.0)	0.9 (0.8, 1.1)	0.9 (0.6, 1.1)	0.9 (0.7, 1.2)	.59
*β*-HCCH^a^	364	1.0 (0.8, 1.3)	1.2 (1.0, 1.6)	1.1 (0.8, 1.5)	1.2 (0.9, 1.7)	.68
HCB^a^	366	1.3 (0.9, 1.7)	1.3 (1.0, 1.8)	1.4 (0.9, 2.3)	1.2 (0.8, 1.8)	.55
PCB 44^a^	326	1.0 (0.7, 1.4)	1.0 (0.7, 1.4)	1.5 (0.8, 2.9)	0.8 (0.5, 1.3)	.12
PCB 49^a^	343	0.8 (0.6, 1.2)	0.8 (0.6, 1.2)	1.2 (0.6, 2.1)	0.7 (0.5, 1.1)	.22
PCB 52^b^	362	0.8 (0.6, 1.2)	0.9 (0.6, 1.2)	1.1 (0.6, 2.0)	0.8 (0.5, 1.2)	.43
PCB 118^c^	342	1.4 (0.9, 2.2)	1.5 (0.9, 2.4)	1.0 (0.5, 2.1)	1.5 (0.8, 2.9)	.41
PCB 138^d^	334	1.5 (1.0, 2.2)	1.8 (1.1,2.8)*	1.1 (0.6, 2.0)	2.2 (1.2,4.0)*	.11
PCB 153^b^	348	1.4 (1.0, 2.1)	1.5 (1.0, 2.4)	1.0 (0.5, 2.0)	1.6 (0.8, 3.0)	.30
PCB 180^e^	284	1.3 (0.9, 2.0)	1.6 (1.1,2.5)*	1.1 (0.6, 2.0)	2.1 (1.2,3.9)*	.11

^a^Adjusted for mother's age at delivery, years living in the United States and marital status; ^b^adjusted for mother's age at delivery, years living in the United States; ^c^adjusted for mother's age at delivery, years living in the United states, marital status, and maternal prepregnancy Body Mass Index; ^d^adjusted for mother's age at delivery and marital status; ^e^adjusted for mother's age at delivery; ^f^ *P*-value for the interaction of breastfeeding history and POP.

Abbreviations: Persistent organic pollutant (POP), sample size (*N*), Hazard Ratio (HR), Confidence Interval (CI), dichlorodiphenyldichloroethylene (DDE), dichlorodiphenyltrichloroethane (DDT), hexachlorocyclohexane (HCCH), hexachlorobenzene (HCB), polychlorinated biphenyl (PCB).*P < .05, **P < .005.

**Table 5:** Adjusted^a^ associations of endocrine-disrupting POPs summarized by principal component analysis and duration of lactation, stratified by breastfeeding history.

	Did not Breastfeed previously			Breastfed previously			
	HR	(95% CI)	*P*	HR	(95% CI)	*P*	*P*_interaction_
Estrogenic^b^	1.0	(0.9, 1.1)	.53	1.0	(0.9, 1.0)	.28	.24
Anti-estrogenic^c^	1.0	(0.9, 1.1)	.79	1.1	(1.0, 1.3)	.03	.13
Androgenic^d^	1.0	(0.9, 1.2)	.54	1.1	(0.9, 1.3)	.28	.67
Anti-androgenic^e^	1.0	(0.9, 1.1)	.63	1.0	(1.0, 1.1)	.49	.42

^a^All model were adjusted for mother's age at delivery, years living in the United States, and marital status. ^b^Estrogenic chemicals include *p*,*p*′-DDE, *p*,*p*′-DDT, *o*,*p*′-DDT, b-HCCH, HCB, PCB 44, PCB 49, PCB 52, PCB153. ^c^Antiestrogenic chemicals include HCB, PCB 118, PCB 138, PCB 153, PCB 180. ^d^Androgenic chemicals include HCB, PCB 118. ^e^Antiandrogenic chemicals include *p*,*p*′-DDE, *p*,*p*′-DDT, *o*,*p*′-DDT, HCB, PCB 118, PCB 138.
